# Attribution theory of poverty and beliefs about charity in Malaysia: an inter-ethnic comparison

**DOI:** 10.3389/fpsyg.2024.1526352

**Published:** 2025-01-15

**Authors:** Abidullah Khan, Muhammad Hakimi Mohd Shafiai, Ghulam Abbas, Syeda Beena Zaidi, Mehboob Ul Hassan

**Affiliations:** ^1^Department of Islamic Economics and Finance, Faculty of Political Sciences, Sakarya University, Sakarya, Türkiye; ^2^Faculty of Economics and Management, National University of Malaysia, Bangi, Selangor, Malaysia; ^3^Department of Business Administration, Sukkur IBA University, Sukkur, Sindh, Pakistan; ^4^Department of Economics, College of Business Administration, King Saud University, Riyadh, Saudi Arabia

**Keywords:** attribution theory, charity, poverty, structuralist beliefs, cash waqf, Malaysia

## Abstract

**Introduction:**

Malaysia as an ethnically diverse country has a history of interethnic inequality and poverty which led to the development of different views about the existence of poverty. Among these ethnic groups, some sympathize with the poor and help them through different charity organizations. However, these charity organizations are mostly unaware of the target donors that can aid their charity funds. Therefore, this study explores the attitudinal differences regarding poverty among Malays, Chinese, and Indians and the demographic groups that can be targeted for donation and social policy development purposes.

**Methodology:**

A survey is conducted in Selangor, where the data through a questionnaire is extracted from the three ethnic groups. Through the quota sampling technique, a sample of 700 is derived. The results are analyzed through ANOVA, regression, and mediation analysis.

**Results:**

The results show that attitudinal differences between Malays and Chinese regarding structuralist beliefs could be observed. Moreover, among Malays, it is found that the relationship between gender, income, education, wealth, and structuralist beliefs is significantly mediated by beliefs about charity. On the other hand, mediation analysis for Chinese identifies gender, education, and income while among Indians gender, education, and wealth are identified as key demographic groups.

**Discussion:**

This study highlights the role of attribution theory to identify the charity donors among the different ethnic groups which was overshadowed by the previous research in Malaysia. The results also hold significance for charity institutions to target the specified groups through their marketing campaigns. Also, based on these results government can reduce the misconception about the existence of poverty and to develop policies that encourage high income groups to support the poor.

## Introduction

Economic disparities between Malaysia's main ethnic groups have existed since British rule and intensified after independence in 1957. Poverty was more prevalent among Malays, primarily in agriculture (Abid et al., 2018), while the Chinese benefitted from control over tin mines (Altas, 1977). Educational inequality emerged post-independence, adding to economic imbalances (Saniman, 2007). Pro-poor policies failed to alleviate Malay poverty, leading to growing discontent, which culminated in ethnic riots following the 1969 election. A national emergency ensued, and a caretaker government introduced the New Economic Policy (NEP) in 1971 (Faaland et al., 1990). This ethnic-based affirmative policy aimed to reduce inter-ethnic income inequalities, primarily benefiting Malays through civil sector quotas and education quotas (Ravallion, 2020; Asadullah et al., 2023). While NEP reduced inter-group inequality, it increased intra-group disparities among Malays (Ravallion, 2001; Khalid, 2014).

To address intra-group inequalities, Malays utilized charities such as sadaqah, zakat, and cash waqf. Similarly, Chinese and Indian communities operated charitable foundations to assist low-income members. However, apart from the government's BSHR scheme targeting low-income groups, most charitable institutions rely on public donations and face challenges in identifying potential donors. Cash waqf, for instance, has been instrumental in human development for the bottom 40% of Malays, where waqif (donors) contributes funds that are managed and invested by a *mutawalli* (manager) according to Islamic principles (Haron et al., 2016; Ali and Markom, 2020; Khan et al., 2022; Amin et al., 2024). Similarly, *zakat* is an obligatory charity (similar to wealth tax) where the one with wealth equivalent to a certain limit is eligible to donate 2.5% of the total wealth to the specific beneficiaries defined in the *Qur'an*.

Despite the good intentions, these institutions face significant inefficiencies due to poor database management and limited identification of potential donors. Similar financial constraints affect non-profit organizations supporting low-income Chinese and Indians, who rely heavily on small donor bases within their ethnic communities (Farouk and Wing, 2019; Ab Samad and Ahmad, 2021).

To address the issues, this study contributes to the current research on poverty in Malaysia in two ways which are ignored by the previous research studies in the same area. First, this study examines the attitudinal differences in poverty among the ethnic groups that have been ignored in the previous studies conducted in Malaysia. Second, this study aims to identify those demographic groups in different ethnic groups that can be targeted for charity campaigns by charity organizations. Therefore, to identify the key donors for charity institutions, it is important to know their attitude toward the poor. In social psychology, the attribution theory of poverty asserts that human beings associate different causes with the condition of the poor. Hence, the framework of this study is inspired by the attribution theory of poverty to identify those individuals who associate structuralist views about poverty.

### Attribution theory of poverty

The attribution theory of poverty has been a significant focus of academic inquiry, particularly from the 1970's to 2010. However, the research is further scant in the later years on this topic. Nevertheless, this study attempts to explore the most relevant literature on the theory, focusing on how demographic groups can be targeted for charitable purposes by examining their beliefs about poverty and charity.

Initially, Simmel (1908), cited in Oorschot and Halman (2000), argued that beliefs about poverty shape welfare policies, with public attitudes determining whether poverty is an individual or societal issue. In the 1970's, studies (Alston and Dean, 1972) found that white Americans predominantly blamed the poor for their circumstances, with younger and more educated respondents being less sympathetic. The attribution theory, developed by Heider (1958), categorizes explanations of behavior into internal (personal) and external (situational) factors, later linked to poverty by Feagin (1972). Feagin identified individualistic, structural, and fatalistic explanations for poverty.

Subsequent research has expanded on these ideas. Feather (1974) found that low-income Australians attributed poverty to societal factors. Furnham (1982) revealed that Labor Party supporters blamed societal factors, while conservatives pointed to individualism. On the other hand, race influences poverty attributions as the difference of opinion could be observed between different races in the United States of America (Hunt, 1996, 2002, 2004, 2016; Bastias et al., 2024). Similar patterns were observed in Russia and Estonia during economic downturns (Stephenson, 2000). Therefore, the first hypothesis of this study is:

H1: There is a significant difference in the attitude of Malays, Chinese, and Indians regarding poverty.

Sociodemographic factors have a significant impact on the beliefs about poverty (Hunt, 1996; Bastias et al., 2019). Different demographic factors are tested against the individualistic and structural explanations of poverty. For instance, a study conducted in Malaysia finds that younger adolescents tended to blame societal factors for poverty, while older ones held both individualistic and structural beliefs (Halik and Webley, 2011), and a similar pattern could be found in the other countries (Bergmann and Todd, 2019; Alcañiz-Colomer et al., 2023). Similarly, women were more likely to endorse individualistic explanations (Halik et al., 2012) contrasting with earlier findings (Kluegel and Smith, 1986; Hunt, 1996, 2004). However, in some recent studies, it is found that women give structural explanations of poverty as compared to their male counterparts (Terol Cantero et al., 2023). Similarly, Khan et al. (2022), in the context of Malays, found that women with structuralist views are more philanthropic, highlighting the role of beliefs in shaping pro-poor attitudes. Therefore, this study hypothesizes that:

H2: Sociodemographic factors have a significant impact on Individualistic beliefs about poverty.
H3: Sociodemographic factors have a significant impact on structural beliefs about poverty.
H4: Sociodemographic factors have a significant impact on fatalist beliefs about poverty.

Additionally, to answer the second objective of the study, this research sheds light on how these beliefs influence people's willingness to help those experiencing poverty. For example, research shows that individuals who attribute poverty to individualistic factors tend to oppose pro-poor policies, whereas those who endorse structural explanations of poverty are more sympathetic toward the poor and favor such policies (Feagin, 1972; Feather, 1974). Similarly, Tuennerman-Kaplan (2001) suggests that people with structural beliefs about poverty are likelier to engage in philanthropy. However, previous studies have not provided empirical evidence to support these claims. This gap is addressed by Khan et al. (2022), who found that women are more likely to hold structuralist views, with their philanthropic nature being a key factor behind this perspective. They also identified the sociodemographic groups who have positive opinions about poverty. However, the study is limited to the Malays ethnic group only. Therefore, this study focuses on inter-ethnic comparison where beliefs about charity mediate the relationship between sociodemographic factors and structural beliefs about poverty.

H5: Beliefs about charity mediate the relationship between sociodemographic factors and structural beliefs about poverty.

To achieve the objectives of this study, the next section presents the methodology, detailing the sampling size and the development of the questionnaire. Following this, the results are reported in the finding section and contextualized within the discussion section. Finally, the conclusion provides a summary of the findings, highlights the study's limitations, and discusses its implications.

## Methodology

### Sampling

This study uses a non-probability sampling technique due to the non-availability of proper databases and limited resources. Selangor is selected as a sample because of its diverse population and the non-probability sampling technique is used to select the respondents for the study. Next, a sample size of 384 is identified based on Krejicie and Morgan's sample size table (Krejicie and Morgan, 1970); however, around 700 questionnaires were distributed.

The quota sampling technique is then applied, dividing the sample into nine districts. To reduce sample bias, first, the sample is categorized based on the population of Malays, Chinese, and Indians in Selangor and then further classified into males and females of each ethnic group. The details regarding sample distribution can be seen in Table 1.

**Table 1:** Sample distribution.

	**Population distribution**						**No. of respondents**					
	**Malay**		**Chinese**		**Indians**		**Malay**		**Chinese**		**Indians**	
**District**	**Male**	**Female**	**Male**	**Female**	**Male**	**Female**	**Male**	**Female**	**Male**	**Female**	**Male**	**Female**
Gombak	196,452	194,208	77,428	70,060	38,916	37,857	28	28	11	10	6	5
Klang	195,774	180,832	116,327	109,098	83,436	81,946	28	26	16	15	12	12
Kuala Langat	68,983	65,360	20,177	18,627	16,955	17,195	10	9	3	3	2	2
Kuala Selangor	73,235	75,819	9,965	8,902	15,363	15,633	10	11	1	1	2	2
Petaling	428,385	424,971	295,584	285,055	97,775	95,269	61	60	42	40	14	14
Sabak Bernam	38,864	39,526	10,737	9,695	2,044	2,053	6	6	2	1	1	1
Sepang	65,411	61,209	16,471	14,409	14,621	13,800	9	9	2	2	2	2
Hulu Langat	290,081	285,404	184,094	171,647	57,576	56,232	41	40	26	24	8	8
Hulu Selangor	66,173	63,910	12,457	11,041	16,190	16,269	9	9	2	2	2	2
**Total**	**1,423,358**	**1,391,239**	**743,240**	**698,534**	**342,876**	**336,254**	**202**	**197**	**105**	**99**	**49**	**48**

The data was collected by approaching the respondents personally. The respondents were informed about the purpose of data collection and their consent was taken before handing over the questionnaire. Later, the incomplete and unfilled questionnaires were discarded, and 520 responses were retained for final analysis.

### Questionnaire and measurement

The questionnaire for this study is adapted from the Khan et al. (2022) study. In their research, four of six independent variables are adopted from previous studies such as Hunt (1996, 2002, 2004), while the authors introduce two. This study considers 11 sociodemographic factors (independent variables) to cover the broader perspective. The factors include ethnicity, gender, marital status, occupation, residence, monthly expenditure, age, dependents, income, wealth, and education.

The dependent variables in their study, i.e., structural, individualistic, and fatalist factors, are adopted from Feather (1974), Furnham (1982), Smith and Stone (1989), and Hunt (2004). The responses were measured on the Likert scale of 1 (Totally disagree) to 6 (Totally agree). Next, the study constructed beliefs about charity variables. This variable is used as a mediating variable in their research. In the current study, the same variable is used for mediation analysis. The reliability analysis for these variables based on Cronbach alpha is reported in Table 2.

**Table 2:** Reliability analysis using Cronbach alpha.

**Individualistic factors (α = 0.747)**
Lack of thrift and proper money management makes people poor.
Lack of effort and laziness by the people push them into poverty.
No attempt at self-improvement among the poor makes them stay in poverty.
Poverty develops due to lack of discipline and responsibility amongst the poor.
Loose morals and habit of drunkenness makes them poor
**Structural factors (**α = **0.771)**
There is a weak or no Government policies to help in alleviating poverty.
Low wages in some businesses and industry creates chances of poverty.
Failure of society to provide good schools makes them poor
Biasness and discrimination against the poor by certain classes push them into poverty
The country's economic instability doesn't allow poor to improve their living standard
**Fatalist factors (**α = **0.856)**
The poor are born with lack of ability and talent that push them into poverty.
Being sick or physically handicapped is one of the causes that doesn't allow one to become rich
Poor are born with lack of intelligence to assist them to drag out of poverty
**Beliefs about charity (0.832)**
I feel sympathy for those in need
I am passionate to help the community financially.
I can afford to help my community financially as much as I can.
I do charity in order to attain happiness
I believe that charity organizations in this country are more effective in helping the needy.
I believe that charities can reduce poverty and income gap amongst the people.

Statistical software packages such as SPSS version 20 and Stata version 17 are used to perform the analysis. Due to the extended sample of this study, factor analysis is performed, and the results are extracted using a principal component method with Eigenvalues of >1. Items carrying a factor loading of >0.5 are retained for each dependent variable. The results are reported in Table 3. Moreover, the factor analysis results of the mediating variable are represented in Table 4.

**Table 3:** Factor analysis of variables derived from attribution theory of poverty.

**Items**	**Factor loadings**		
	**Structuralist**	**Individualistic**	**Fatalist**
Lack of thrift and proper money management makes people poor.		0.771	
Lack of effort and laziness by the people push them into poverty.		0.807	
No attempt at self-improvement among the poor makes them stay in poverty.		0.606	
Poverty develops due to lack of discipline and responsibility amongst the poor.		0.614	
Loose morals and habit of drunkenness makes them poor.		0.529	
There is a weak or no Government policies to help in alleviating poverty.	0.699		
Low wages in some businesses and industry creates chances of poverty.	0.717		
Failure of society to provide good schools makes them poor.	0.567		
Biasness and discrimination against the poor by certain classes push them into poverty.	0.749		
The country's economic instability doesn't allow poor to improve their living standard.	0.721		
The poor are born with lack of ability and talent that push them into poverty.			0.863
Being sick or physically handicapped is one of the causes that doesn't allow one to become rich.			0.871
Poor are born with lack of intelligence to assist them to drag out of poverty			0.878

**Table 4:** Factor analysis of beliefs about charity.

**Items**	**Factor loadings**
I feel sympathy for those in need	0.786
I am passionate to help the community financially.	0.842
I can afford to help my community financially as much as I can.	0.841
I do charity in order to attain happiness	0.821
I believe that charity organizations in this country are more effective in helping the needy.	0.559
I believe that charities can reduce poverty and income gap amongst the people.	0.588

## Finding

This section reports the results of the hypotheses of this study. However, before reporting the main findings of this study, descriptive analysis was performed to develop an understanding of the distribution of the sample of the study. In this regard, Table 5 shows the descriptive characteristics of the sample. Most respondents were Malays, comprising 57.9% of the sample, followed by Chinese (27.7%) and Indians (14.4%). About half of the respondents were married, and approximately the same percentage of respondents were male. Moreover, most respondents had undergraduate degrees (38.7%), whereas about 42.1% had monthly income between RM 2001-4000. The majority had no wealth possession, and most respondents reported a deficit in what they earned and what they spent. Most respondents aged between 25 and 30 years and ~74% lived in Selangor's urban areas. Regarding occupation, half of the sample worked in the private sector at the time of the data collection, and most respondents reported no dependents.

**Table 5:** Descriptive statistic of socio-demographic factors.

**Socio-demographic factor**	**Frequency**	**Percentage**
**Ethnicity**		
Malay	301	57.9
Chinese	144	27.7
Indians	75	14.4
**Marital status**		
Single	208	40.0
Married	277	53.3
Widowed	20	3.8
Separated	15	2.9
**Gender**		
Male	267	51.3
Female	253	48.7
**Level of education**		
No formal education	8	1.5
Primary	3	0.6
Secondary	108	20.8
Diploma	153	29.4
Degree	199	38.3
Masters	43	8.3
Ph. D.	6	1.2
**Monthly Income**		
Below 2,000	165	31.7
2,001–4,000	219	42.1
4,001–6,000	86	16.5
6,001–8,000	33	6.3
8,001–10,000	7	1.3
Above 10,000	10	1.9
**Do you have inherited wealth?**		
No	380	73.1
Yes	140	26.9
**Is your monthly expenditure more than your income?**		
No	375	72.1
Yes	145	27.9
**Age**		
18–24	68	13.1
25–30	171	32.9
31–36	153	29.4
43–48	65	12.5
43–48	38	7.3
49–54	19	3.7
55–60	5	1.0
Above 60	1	0.2
**Residence**		
Urban	386	74.2
Rural	134	25.8
**Occupation**		
Civil Servant	135	26.0
Private Sector	265	51.0
Self Employed	87	16.7
Un-Employed	33	6.3
**Excluding yourself, how many dependents do you have?**		
0	183	35.2
1	58	11.2
2	89	17.1
3	67	12.9
4	55	10.6
5	33	6.3
6	21	4.0
7	4	0.8
8	9	1.7
10	1	0.2

Table 6 presents the results of a one-way analysis of variance (ANOVA) conducted to assess the variations in attitudes toward poverty among various ethnic groups (Fisher 1970). Regarding individualistic beliefs, the Chinese exhibit a higher mean score (4.2722) than other ethnic groups. Nevertheless, no statistically significant difference is observed among the mean scores of all ethnic groups (*F* = 0.43, *p* > 0.05). This suggests that these ethnicities share similar beliefs regarding the individualistic aspects of poverty. Regarding structuralist beliefs, the Chinese have the highest mean score (4.4514), signifying their belief that poverty is primarily attributable to a lack of opportunities and external factors. A significant difference is observed between the mean scores of the Chinese and Malays, while Malays and Indians hold similar views about poverty in this context. Whereas, fatalistic beliefs about poverty, there is a substantial difference (*F* = 29.22, *p* < 0.01) among the group means of all ethnicities. Compared to Malays, both Chinese and Indians are more likely to endorse fatalistic beliefs about poverty, attributing it to bad luck as a prominent reason. It can also be observed that all ethnicities agree on the existence of poverty due to individualistic factors as well as structuralist factors.

**Table 6:** ANOVA summary.

**Group**	**Mean**	**(1)**	**(2)**	**(3)**
**Individualistic belief about poverty**				
(1) Malay	4.2046	-	ns	ns
(2) Chinese	4.2722		-	ns
(3) Indians	4.1786			-
*F* = 0.43, *p* = 0.6491			
**Structuralist belief about poverty**				
(1) Malay	4.1541	-	yes	ns
(2) Chinese	4.4514		-	ns
(3) Indians	4.2826			-
*F* = 6.44, *p* = 0.0017			
**Fatalistic belief about poverty**				
(1) Malay	3.0366	-	yes	yes
(2) Chinese	3.9513		-	yes
(3) Indians	3.5068			-
*F* = 29.22, *p* = 0.0000			

For each dependent variable, the groups are ranked according to their means. There are three possible group mean comparisons in which “ns” represents no significant difference between one group mean compared to the other, whereas “yes” represents that two groups are significantly different.

A series of Ordinary Least Squares (OLS) regression models were conducted to test hypotheses 2, 3, and 4, which investigate how different ethnic groups influence views on poverty while controlling demographic factors. The results of these regression models, specifically focusing on individualistic, structuralist, and fatalistic beliefs about poverty, are summarized in Table 7.

**Table 7:** Regression summary.

	**Individualistic belief**		**Structuralist belief**		**Fatalistic belief**	
	**(1)**	**(2)**	**(1)**	**(2)**	**(1)**	**(2)**
**Ethnic**						
Chinese	0.065	−0.002	0.277^***^	0.199^***^	0.918^***^	0.956^***^
	(0.084)	(0.077)	(0.084)	(0.076)	(0.115)	(0.112)
Indian	−0.022	−0.143	0.139	0.075	0.483^***^	0.597^***^
	(0.108)	(0.098)	(0.107)	(0.097)	(0.148)	(0.142)
**Gender**						
Male	−0.111	−0.038	−0.099	−0.032	0.725^***^	0.658^***^
	(0.073)	(0.066)	(0.072)	(0.065)	(0.100)	(0.096)
**Marital status**						
Married	0.089	0.169^**^	0.100	0.100	0.159	0.245^**^
	(0.076)	(0.085)	(0.075)	(0.084)	(0.104)	(0.123)
Widow	0.360^*^	0.389^**^	−0.152	−0.188	0.075	0.207
	(0.199)	(0.184)	(0.198)	(0.182)	(0.273)	(0.265)
Separated	0.107	0.281	−0.257	−0.177	−0.428	−0.351
	(0.215)	(0.198)	(0.214)	(0.196)	(0.295)	(0.285)
**Occupation**						
Private	−0.004	0.015	−0.057	−0.066	−0.299^**^	−0.314^***^
	(0.085)	(0.079)	(0.084)	(0.078)	(0.116)	(0.113)
Self-emp.	0.003	0.019	−0.041	−0.061	−0.203	−0.163
	(0.115)	(0.105)	(0.114)	(0.104)	(0.158)	(0.151)
Unemployed	0.123	0.117	−0.004	−0.081	0.026	−0.071
	(0.152)	(0.143)	(0.151)	(0.141)	(0.208)	(0.206)
**Resident**						
Rural	0.029	0.043	0.075	0.064	0.0045	−0.034
	(0.084)	(0.076)	(0.084)	(0.076)	(0.116)	(0.110)
**Monthly exp**.						
Yes	0.138^*^	0.156^**^	0.038	0.057	0.062	0.065
	(0.081)	(0.073)	(0.080)	(0.072)	(0.111)	(0.106)
Age		−0.006		−0.029		−0.024
		(0.026)		(0.025)		(0.037)
Dependents		−0.035^*^		−0.003		−0.028
		(0.020)		(0.019)		(0.029)
Income		0.041^*^		0.007		−0.226^***^
		(0.021)		(0.021)		(0.031)
Wealth		0.245^***^		0.543^***^		0.156
		(0.069)		(0.068)		(0.099)
Education		−0.247^***^		−0.168^***^		0.093^**^
		(0.026)		(0.025)		(0.037)
_cons	40.143^***^	40.957^***^	40.172^***^	40.709^***^	20.744^***^	30.018^***^
	(0.097)	(0.172)	(0.096)	(0.170)	(0.133)	(0.247)
Observations	520	520	520	520	520	520
R-squared	0.021	0.214	0.04	0.234	0.209	0.296

In this table, the Malay group is considered a reference group, and in the gender predictor, female is regarded as a reference to compare the mean values with the other categories of respective independent variables. Standard errors are in parentheses, and the significance of variables is represented with ^***^p < 0.01, ^**^p < 0.05, ^*^p < 0.1.

### Individualistic belief

In the initial model, monthly expenses and marital status were statistically significant at 10%. This suggests that, when comparing Malay, Indian, and Chinese females, there is a modest inclination to believe that poverty is mostly attributed to a lack of education and abilities. However, when demographic factors are considered in the second model of individualistic beliefs, the significance of the coefficients for marital status and monthly expenses has improved. Additionally, inherited wealth substantially impacts the development of individualistic beliefs. Education plays a crucial role in shaping Individuals' perspectives, especially regarding socioeconomic status. Specifically, females with limited educational opportunities who possess inherited wealth tend to adopt more individualistic ideologies that perpetuate the cycle of poverty than males.

### Structuralist belief

In the second model of structuralist beliefs, the coefficient for Chinese ethnicity is positively and statistically significant at the 1% level. The coefficient of inherited wealth is also statistically significant at the 1% level. Education has a notable negative impact on structural beliefs. It may be inferred that, when compared to individuals of other ethnic backgrounds, Chinese females with higher levels of education are less likely to support the socioeconomic element as a cause of poverty. On the other hand, women with higher degrees of inherited wealth seem to have a stronger inclination toward embracing the structuralist perspective on poverty compared to men. The predominant tendency is to attribute poverty mostly to societal factors or the absence of income-generating alternatives.

### Fatalist belief

In the initial model, when comparing Malay ethnicity to Chinese and Indian ethnicities, the latter two groups exhibit a statistically significant positive influence on fatalistic attitudes at the 1% level. The coefficient of gender shows a positive and statistically significant relationship at the 1% level. Males of Chinese and Indian descent exhibit a higher tendency to endorse fatalistic ideas regarding poverty compared to females. In contrast, the occupation variable shows a significant negative association with fatalistic attitudes at a 5% level. Chinese and Indian males engaged in private-sector employment are less likely to endorse fatalistic beliefs regarding poverty.

After accounting for demographic factors, the second model enhances the relevance of private sector occupation. The analysis reveals a statistically significant association between marital status and fatalistic belief at a 5% level. Married males tend to hold the opinion that certain individuals are predetermined to experience poverty. The data also indicates a statistically significant negative relationship between income and fatalistic thoughts about poverty at the 1% level. This suggests that individuals with lower income levels place greater emphasis on fatalistic beliefs about poverty. The coefficient of education has exhibited a positive relationship in this context, with a significance level of 5%. The presence of inherited wealth significantly positively influences fatalistic beliefs, as determined at a 5% level.

Table 8 explains the role of Charity (the mediator variable M) in the relationship between demographic factors (independent variable X) and structural beliefs about poverty (the dependent variable Y) for the Malay ethnic group. The results show that the total and direct effects of gender on the structuralist belief about poverty are insignificant, respectively. However, when we included charity as a mediating variable in our model, the relationship between gender and structuralist belief became significant. Thus, the indirect effect of gender on structuralist beliefs through charity is statistically significant. These findings indicate that gender does not directly affect the structural belief about poverty rather, but through charity, it does.

**Table 8:** Mediation analysis summary for Malays (ethnic group 1).

**Effect**	**Path**	**Coefficient**	**S.E**.	***z*-value**	***p*-value**
Total	Gender → Structuralist	−0.598	0.087	−6.820	0.000
Indirect	Gender → Charity → Structuralist	−0.574	0.075	−7.590	0.000
Direct	Gender → Structuralist	−0.023	0.054	−0.430	0.668
Total	Education → Structuralist	−0.250	0.031	−7.930	0.000
Indirect	Education → Charity → Structuralist	−0.240	0.027	−8.730	0.000
Direct	Education → Structuralist	−0.009	0.020	−0.470	0.639
Total	Income → Structuralist	0.174	0.027	6.330	0.000
Indirect	Income → Charity → Structuralist	0.161	0.023	6.820	0.000
Direct	Income → Structuralist	0.013	0.016	0.780	0.438
Total	Wealth → Structuralist	0.604	0.090	6.680	0.000
Indirect	Wealth → Charity → Structuralist	0.531	0.077	6.830	0.000
Direct	Wealth → Structuralist	0.073	0.055	1.340	0.181

Similarly, the relationship between education and income and structuralist factors and wealth and structuralist factors is also mediated by charity. With increased income levels, Malays become more sympathetic toward the poor because of their philanthropic nature. Conversely, with the decreased education level, Malays have become more sympathetic toward the poor because of their philanthropic nature. The results are aligned with the study of Khan et al. (2022), which suggests that beliefs about charity mediate the relationship between these variables.

Table 9 shows the mediation analysis results for the Chinese ethnic group. The results show that the beliefs about charity mediate the relationship between gender and structuralist beliefs and education and structuralist beliefs. It means that Chinese males are more concerned about people experiencing poverty because of their strong philanthropic beliefs. However, this result contrasts with the Malay group, where the same result holds for the female group. Similarly, the low-level education holders are more concerned about the poor and hold societal factors responsible for poverty because they are more philanthropic.

**Table 9:** Mediation analysis summary for Chinese (ethnic group 2).

**Effect**	**Path**	**Coefficient**	**S.E**.	***z*-value**	***p*-value**
Total	Gender → Structuralist	0.739	0.129	5.710	0.000
Indirect	Gender → Charity → Structuralist	0.002	0.007	0.340	0.000
Direct	Gender → Structuralist	0.736	0.129	5.690	0.738
Total	Education → Structuralist	−0.447	0.058	−7.630	0.000
Indirect	Education → Charity → Structuralist	−0.001	0.007	0.110	0.000
Direct	Education → Structuralist	−0.447	0.059	−7.580	0.912
Total	Income → Structuralist	−0.235	0.045	−5.190	0.000
Indirect	Income → Charity → Structuralist	0.001	0.003	0.170	0.866
Direct	Income → Structuralist	−0.236	0.045	−5.210	0.000
Total	Wealth → Structuralist	0.632	0.133	4.730	0.000
Indirect	Wealth → Charity → Structuralist	0.002	0.019	0.110	0.909
Direct	Wealth → Structuralist	0.630	0.135	4.670	0.000

Table 10 reports the mediation analysis results of the Indian ethnic group. The results show that the relationship between gender, education, income, and wealth with structuralist beliefs is mediated by beliefs about charity. Like the Chinese group, Indian males hold more structuralist views about poverty because of their strong philanthropic beliefs. Also, with a decrease in education level, the structuralist beliefs strengthen due to the strong charitable beliefs of the Indian ethnic group. Like Malays, with the increase in income and wealth, the beliefs of the Indians become more structuralist due to their strong beliefs about doing charity.

**Table 10:** Mediation analysis summary for Indians (ethnic group 3).

**Effect**	**Path**	**Coefficient**	**S.E**.	***z*-value**	***p*-value**
Total	Gender → Structuralist	0.387	0.164	2.360	0.018
Indirect	Gender → Charity → Structuralist	0.345	0.138	2.490	0.013
Direct	Gender → Structuralist	0.042	0.095	0.440	0.658
Total	Education → Structuralist	−0.239	0.051	4.630	0.000
Indirect	Education → Charity → Structuralist	−0.243	0.046	5.260	0.000
Direct	Education → Structuralist	−0.004	0.038	−0.110	0.913
Total	Income → Structuralist	0.061	0.050	−1.210	0.225
Indirect	Income → Charity → Structuralist	0.087	0.043	−2.010	0.045
Direct	Income → Structuralist	0.025	0.028	0.890	0.375
Total	Wealth → Structuralist	0.507	0.166	3.040	0.002
Indirect	Wealth → Charity → Structuralist	0.329	0.140	2.350	0.019
Direct	Wealth → Structuralist	0.177	0.096	1.840	0.066

## Discussion

This study primarily aimed to test the attribution theory in the context of Malaysia and to identify the charity donors for the charity institutions in Malaysia. Therefore, a stepwise approach is used to identify those more sympathetic toward the poor and then identify the sociodemographic groups the charity institutions can target for their campaigns. Hence, to identify the donors based on ethnicity, this study first performed ANOVA analysis and found that all ethnic groups hold similar views regarding individualist and structural beliefs about poverty. Similar results have been achieved by Hunt (2004) which showed that American holds both individualist and structuralist views about poverty. The author termed it as dual consciousness. Similarly, Khan et al. (2022) also report similar patterns among the Malays.

Based on the regression analysis, it is identified that Chinese women with higher levels of education are less likely to suggest structural factors of poverty as the main cause. However, those females with high levels of wealth tend to support that poverty exists because of societal neglect. Further, studies claim that the strength of beliefs about poverty depends on one's tendency toward beliefs about charity. In other words, those who believe that poverty exists because of societal neglect are more likely philanthropic (Feagin, 1972; Feather, 1974; Tuennerman-Kaplan, 2001). The empirical evidence of such claims has been tested in Malaysia and found that Malays hold structural beliefs because of their philanthropic nature (Khan et al., 2022). Therefore, this study tested the mediating role of beliefs about charity on the relationship between the independent variables (sociodemographic factors) and the dependent (structural factors) variables.

This study finds that the relationship between gender, education, income, wealth (independent variables), and structural factors is mediated by beliefs about charity. The results partially align with the claims of Khan et al. (2022) where gender and education were the identified independent factors in mediation analysis. In this study, it is identified that females, those with low levels of education, higher income, and no wealth, could be targeted by charity institutions for their charity campaigns. On the other hand, Chinese male with a lower level of education can be targeted by Chinese charity institutions for their donation campaigns. Like Chinese, Indian males with structural beliefs are more inclined toward charity. Also, like Malays, those with lower levels of education, high incomes, and no wealth could be the key targets of Indian charity institutions in identifying target groups for charity campaigns.

## Conclusion

This study guides charity institutions to design marketing strategies by considering the specific demographic groups identified in this study. In this regard, it is identified that all three ethnic groups i.e., Malays, Chinese, and Indians hold the same views about the existence of poverty. Further, it is found that Malay and Indian females, and those with lower education, high levels of income, and no wealth can be the main targets for the charity institutions. On the other hand, Chinese males, and those with lower education are the potential donors for the Chinese charity institutions. The study also holds importance for the government to develop policies that can address the misconception about poverty and pro-poor policies. For instance, the development of educational plans that aim to address the misconception about poverty across all ethnic groups and executed at higher schooling levels. Also, the government can encourage high-income groups by providing incentives to support charity programs that aim at reducing inequality and poverty.

Nevertheless, the study also has some limitations. First, due to no access to a proper database, this study is based on non-probability sampling techniques, which are statistically less sound than the probability sampling technique. Second, due to budget constraints, the data is only collected from one state of the country, i.e., Selangor. Nevertheless, future researchers can develop their sample based on the Household Income and Expenditure Survey Database if they can access it from the Department of Statistics in Malaysia. Moreover, the research on wealth inequality is more eminent in Malaysia nowadays, it is an opportunity for the future researcher to replicate the same study by using attribution theory in the context of wealth inequality. This study also identifies key demographic groups, providing a foundation for further research aimed at developing pro-poor government policies.

## Data Availability

The raw data supporting the conclusions of this article will be made available by the authors, without undue reservation.
